# A case study evaluating the use of clozapine in depression with psychotic features

**DOI:** 10.1186/1744-859X-5-20

**Published:** 2006-11-29

**Authors:** Premkumar Jeyapaul, Ray Vieweg

**Affiliations:** 1 Wimborne and Purbeck community mental health team, Oakley bungalow 15 Oakley Lane, Canford Magna, Wimborne, Dorset, BH21 1SF, UK; 2 The Beeches, St James Hospital, Portsmouth, Hampshire, PO4 8LD, UK

## Abstract

The purpose of this case study was to use an evidence based medicine approach to work through an unusual way of treating a common problem. We looked at an example of an in-patient with severe refractory psychotic depression who had been resistant to treatment with a combination of antidepressant, antipsychotics, mood stabiliser, and concomitant ECT therapy.

We then undertook a literature search for the use of clozapine in a patient with severe refractory depression.

Although the resulting evidence was low level and thin, we felt on balance that a trial of clozapine was justified.

We used a BPRS inventory to monitor her mood prior to commencing clozapine. Her mood and functional abilities were monitored as her clozapine was titrated upwards.

Our patient showed a significant improvement in mood and functional abilities and a reduction in her BPRS score during this period. Her symptoms improved to the point where she was successfully discharged home on a combination of clozapine and an antidepressant.

The improvement was sustained for a further two years.

We thought this was an important case to highlight the limited evidence in using this successful form of treatment for a common clinical problem and that further research in this area was needed.

## Background

We present a case study using the Evidence Based Medicine approach and have attempted to work through a common problem. The aim of the process was to underpin our clinical decision with relevant research evidence.

### Case

A 39-year-old married housewife with 2 children aged 16 and 12 years was electively admitted for treatment of worsening depression.

She had a 5-year history of recurrent severe depressive episodes; there had been no history of mental health problems prior to this. She had been an in-patient for most of the last 5 years, and had required one-to-one nursing on one admission because of self-harming behaviour, which included cutting and trying to set herself on fire.

She had been raped at the age of 12 years; however, prior to her first episode of depression she had a well-adjusted pre-morbid personality, having not had any symptoms suggestive of post traumatic stress disorder prior to her history of depression. A diagnosis of post traumatic stress disorder had been considered, however, rejected because her depressive affective symptoms dominated her clinical presentation, and she did not suffer flashbacks to her index traumatic episode. Other diagnoses that merited consideration included schizoaffective disorder and bipolar disorder, however, she did not suffer from first rank symptoms of schizophrenia or hypomanic/manic episodes which excluded her respectively from both of these diagnoses according to ICD-10.

The depressive symptoms followed soon after a triggering event of a horse-riding accident from which she suffered concussion. A CT scan at the time was reported as normal.

She had a family history of mental disorder, with a sister who suffered from schizophrenia.

During this present admission she had experienced 5-month deterioration in mood. She had a 1-week period of insomnia and increasing suicidal ideation. There was no history of alcohol/substance misuse or any medical problems. She had been receiving ECT treatment twice weekly in the community for the 4 months preceding the admission. The ECT continued after she was admitted. Her medication was:

Lithium carbonate 1000 mg once daily

Mirtazapine 60 mg at night

Olanzapine 20 mg at night

Chlorpromazine 50 mg at night

On MSE, she had psychomotor retardation with poor eye contact and a constant rocking motion. Her affect was melancholic. There was no formal thought or perceptual disorder, or evidence of cognitive impairment.

On 13/8/01 she was started on a 4-day course of dexamethasone, which is an unusual but published treatment for resistant depression (Dexamethasone augmentation in treatment resistant depression *Acta Psychiatr Scan *1997; 95 58–61). There were no obvious beneficial effects, and she still felt low in her mood, now with a blunted affect. Her speech was slower and more monotonous. She had decreased motivation. She had worsening suicidal ideation.

On 19/8/01 the patient became very agitated and started lashing out. Several staff members were needed to restrain her and she was sedated with IM lorazepam. The following day she reported hearing voices of her rapists saying derogatory comments to her.

The patient's mirtazapine, chlorpromazine and olanzapine medication were stopped and she was started on haloperidol and amitriptyline. Two days later, she started experiencing second person auditory hallucinations of the rapist who assaulted her in her childhood. She became more restless and agitated. Her suicidal ideation increased and she had difficulty thinking clearly. She continued to receive ECT treatment and her medication was increased to 200 mg amitriptyline and 40 mg of haloperidol.

#### Formulating an Evidence Based Medicine question

We felt it was important to formulate an EBM question that could be researched in view of possible treatment approaches that we could offer. We had already tried her on a variety of medical treatments, which had limited benefit.

The question formulated was as follows: '*In a patient who has depression with psychotic symptoms, is the use of clozapine and an antidepressant more beneficial than standard treatments for psychotic depression, in improving mood and psychotic symptoms.*'

#### Literature search

The literature search that was conducted used the following databases:

Cochrane Database, ACP Journal Club, CCTR, 1986–2002

Medline 1966–2002.

EMBASE 1993–1996.

PSYCHINFO 1887–2002

The manufacturers of clozapine were also contacted.

The keywords used were:

1 'Depression/Depressive disorder/mood disorder/affective disorder/Psychosis/Psychotic'

2 The above keywords were combined with 'clozapine and antidepressant agents'.

The search was originally limited to the years 1986–2002, English language, and human research (however, the limitations were not valid in Cochrane, ACP, DARE, CCTR). However, an up to date review on the above databases up to 2006 was conducted on follow up of the patient which yielded no further pertinent papers.

The original search initially yielded 147 articles but on further inspection only 8 were thought to be pertinent to the question. The lists of references for these papers were also reviewed.

## Critically appraising the evidence

As we can see from the table above the evidence is limited. The only evidence specific for the question is limited to low-level evidence with only case study/series evidence. The higher quality evidence is limited by being non-specific, especially with regards to the case that we are considering, as the evidence is looking at a heterogeneous population with conditions including schizophrenia, bipolar disorder and psychotic depression, often without comparison groups, and varying outcome measures.

## Summary of the evidence

The following evidence [1-3,7] [see table 1], all conducted retrospective chart/case series that suggested that clozapine was effective in improving affective symptoms, and in improving psychotic symptoms across a range of diagnoses from schizophrenia to depression with psychotic symptoms, however a major limitation to these studies is that they tended to focus on a heterogeneous population, with a minority of patients that had depression with psychotic symptoms. There was also a lack of comparison study groups in these trials.

**Table 1:** Key Papers identified from the literature search

Author	Date	Description	Type of Study	Outcome	Limitations
McElroy SL, Dessain EC, Pope HG Jr, Cole JO, Keck PE Jr, Frankenberg FR, Aizley HG, O'Brien S [1]	1991	Clozapine in the treatment of Psychotic mood disorders, schizoaffective disorder and schizophrenia.	Retrospective Chart analysis of patients with 39 schizophrenia, 25 schizoaffective disorder and 14 bipolar disorder with psychotic features	Clozapine was shown to be useful in the treatment of patients with schizoaffective disorder or psychotic mood disorder who are treatment resistant or intolerant of side effects.	There was no standardisation of treatment given prior to clozapine administration. There was no comparison group or long term follow up of patients.
Banov MD, Zarate CA Jr, Tohen M, Scialabba D, Wines JD Jr, Kolbrener M, Kim JW, Cole JO. [2]	1994	Clozapine therapy in refractory affective disorders: polarity predicts response in long-term follow up.	Retrospective Review of 193 Case Notes of 52 Bipolar Disorder, 81 schizoaffective disorder, 14 unipolar depression, 40 schizophrenia, and 6 other disorder.	Clozapine is an efficacious and well-tolerated therapy for refractory affective illness. Manic symptomatology predicts a more favourable response than depression.	Heterogeneous groups studied, not standardised for demographics, no comparison group for treatment with clozapine, no knowledge of prior treatment given.
Collaborative Working Group on Clinical Trial Evaluations.[3]	1998	Evaluating the usefulness of atypical antipsychotics in reducing suicidality in schizophrenic patients and their use in affective disorders	Review	Atypical antipsychotics may be used as an adjunctive medication or an alternative to mood stabilizers in patients with affective disorders	Not specific to clozapine and not evaluating controlled trials.
Suppes T, Webb A, Paul B, Carmody T, Kraemer H, Rush AJ.[4]	1999	Clinical Outcome in a Randomised 1 year trial of Clozapine versus treatment as usual for patients with treatment resistant illness and a history of mania	Prospective randomised trial 38 patients with schizoaffective disorder and bipolar disorder.	Showed that clozapine had independent mood stabilising properties.	Looks at heterogeneous population of Not specific to psychotic depression.
Ranjan R, Meltzer HY.[5]	1996	Acute and Long term Effectiveness of Clozapine in treatment -resistant psychotic depression	Case Series Only 3 cases studied.	In all 3 cases, clozapine treatment was associated with significant improvement in both affective and psychotic symptoms. Maintenance in remission was sustained over many years	Only 3 cases studied, there was no comparison group.
Zarate CA Jr, Tohen M, Baldessarini RJ.[6]	1995	Clozapine in Severe Mood Disorders.	Systematic Review	Clozapine appears to be effective and well tolerated in the short term and maintenance of severe or psychotic mood disorders.	Heterogeneous analysis of different trials including case series, retrospective analysis and open label trials. No double blind studies specific to the treatment with clozapine. Outcome measures varied according to the trial used.
Naber D, Holzbach R, Perro C, Hippius H.[7]	1992	Clinical Management of Clozapine Patients in Relation to Efficacay and Side-Effects.	Retrospective analysis of 644 medical charts, patients who were given clozapine after standard neuroleptic treatment had failed.	Efficacy of clozapine was satisfactory in 55–72% of patients with organic psychosis, mania, psychotic depression or Parkinson's disease.	Majority of patients had schizophrenia and schizoaffective disorder only 54 had psychotic depression. The study tended to focus on the side effect profile of clozapine in schizophrenia
Dassa D, Kaladjian A, Azorin JM, Giudicelli S.[8]	1993	Clozapine in the Treatment of Psychotic Refractory Depression.	Case Study	Depressive and psychotic features improved after the administration of clozapine suggesting that clozapine could be efficient in psychotic refractory depression	Only one case studied.

A randomised prospective trial examining clozapine versus treatment as usual [4] [see table 1] performed in a patient population with schizoaffective disorder or bipolar disorder, the results of which suggested that clozapine had independent mood stabilising effects. However, it did not have a sub-group of patients with depression with psychotic symptoms, and therefore it was not suitable for formulating an answer to our question.

A systematic review [6] [see table 1] of retrospective studies, open label trials, some of which have been included in the literature review, showed clozapine was useful in the short term and maintenance of symptoms of patients with severe psychotic mood disorders. However, the limitations again were that the trials reviewed had a heterogeneous population with outcome measures that varied according to the trial studied. There was no double blind comparison trial of clozapine in depression with psychotic symptoms.

Case study and case series [5,8] work show that clozapine treatment was useful in a select number of cases of refractory depression with psychotic features that had failed to respond to conventional treatment including ECT treatment and other neuroleptic medication. This was very specific to the case that we were studying, however, the evidence was at a low level in the hierarchy.

## Intervention

Although the evidence is thin, our patient's symptoms were severe and other treatment for depression had failed. On balance, we felt that a trial of clozapine was justified. On 7/9/01 after a discussion with her and her husband, including an explanation that clozapine treatment, would be used outside its license, it was decided that she should commence clozapine. Other medication at the time was (daily dose):

Amitriptyline 200 mg

Haloperidol 40 mg

Lithium 1000 mg

Haloperidol was decreased, ECT treatment was stopped and clozapine was started on the 28/9/01, and gradually titrated upwards monitoring her mental state and side effects.

A BPRS rating scale performed four days prior to commencing on clozapine was 63. She had marked decrease in emotional contact, very confused thought processes, visible nervousness and tension. Her mood was very low; she had gross psychomotor retardation and a blunted affect.

Over the subsequent few weeks she continued experiencing low mood, ongoing suicidal ideation, and more confusion and had difficulty completing sentences. She experienced difficulty waking up and problems with her short-term memory.

Four weeks later the BPRS score was 39. She was less emotionally withdrawn and was interacting better with those around her. She was improved in mood, her affect was more reactive and there was considerable improvement in her psychomotor functioning. She still exhibited some thought confusion and showed less physical tension and nervousness. She was now experiencing somatic hallucinations of someone touching her but the auditory hallucinations were less intense.

**Figure 1:**
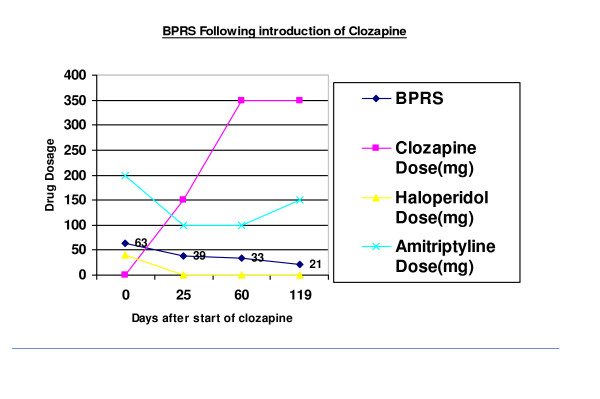


She continued to improve and was now able to go on some home leave with her husband. Two months after starting clozapine there was a global improvement in her BPRS 33 [see figure 1], however, she still appeared low in her mood. However, she experienced hypersalivation and her amitryptiline was increased because of its antimuscarininic effects. She was sleeping well with no difficulties getting up in the morning and there was less suicidal ideation.

After four months of clozapine treatment, her BPRS score was 21 [see figure 1] and she scored minimally on all criteria measured. She was now having extended periods of home leave on her own and was able to laugh and joke with other staff members. She no longer experienced any somatic/auditory hallucinations. She was able to resume other activities at home including domestic duties and shopping.

## Conclusion

The evidence based medicine approach provides a framework with which to use evidence to support clinical practice. It guides the clinician in making a decision about treatment. However, with regard to the question that was asked, the evidence supporting use of clozapine was rather limited. The approach allows clinicians to use relatively weak evidence with other clinical skills. It necessitates the use of innovative practice at times, and enables clinicians to contribute to research ideas and development. EBM reviews such as this may contribute to further research ideas.

In this case clozapine made a significant improvement in clinical state. There were many uncontrolled factors that could have contributed to the overall improvement but in this patient the resistance to all previous treatments suggests that clozapine use was responsible for the improvement. It could be argued that the use of amitriptyline could have contributed; however, it had been used on several previous occasions with no benefit.

We thought it would be interesting to follow up the patient to see if the benefits of clozapine treatment were sustained. The patient had an admission for another depressive episode almost two years later, and was then discharged on clozapine 150 mg in the morning and 300 mg at night. She was subsequently reviewed in the outpatient clinic regularly and has remained well.

Another literature search (of the same original databases) yielded no further evidence supporting the use of clozapine in depression with psychotic symptoms, other than a single case report [9] which described a case of a 48 year old woman with refractory depression who responded well to a combination of clozapine and maprotilline after ECT and other antidepressants failed to work, however this did not address our question as the case did not have psychotic symptoms.

Referring back to the original question '*In a patient with depression with psychotic symptoms, is the long term use of clozapine and antidepressant more beneficial than standard, second and third line treatment for psychotic depression, in improving mood and psychotic symptoms*', there remains much uncertainty and we would support further research in this area, allowing us to give more than a reserved and qualified affirmation.
